# Effects of Aerobic-, Anaerobic- and Combined-Based Exercises on Plasma Oxidative Stress Biomarkers in Healthy Untrained Young Adults

**DOI:** 10.3390/ijerph17072601

**Published:** 2020-04-10

**Authors:** Achraf Ammar, Khaled Trabelsi, Omar Boukhris, Jordan M Glenn, Nick Bott, Liwa Masmoudi, Ahmed Hakim, Hamdi Chtourou, Tarak Driss, Anita Hoekelmann, Kais El Abed

**Affiliations:** 1 Institute of Sport Science, Otto-von-Guericke-University Magdeburg, 39106 Magdeburg, Germany; anita.hoekelmann@ovgu.de; 2 Institut Supérieur du Sport et de l’Education Physique de Sfax, Université de Sfax, Sfax 3000, Tunisia; trabelsikhaled@gmail.com (K.T.); omarboukhris24@yahoo.com (O.B.); liwa.masmoudi@yahoo.fr (L.M.); kais.elabed@gmail.com (K.E.A.); 3 Research Laboratory: Education, Motricité, Sport et Santé, EM2S, LR19JS01, High Institute of Sport and Physical Education of Sfax, University of Sfax, Sfax 3000, Tunisia; 4 Activité Physique, Sport et Santé, UR18JS01, Observatoire National du Sport, Tunis 1004, Tunisia; 5 Neurotrack Technologies, Redwood City, CA 94063, USA; jordan@neurotrack.com (J.M.G.); nick@neurotrack.com (N.B.); 6 Exercise Science Research Center, Department of Health, Human Performance and Recreation, University of Arkansas, Fayetteville, AR 72701, USA; 7 Clinical Excellence Research Center, Department of Medicine, Stanford University School of Medicine, Stanford, CA 94305, USA; 8 Laboratory of Pharmacology, Faculty of Medicine, Sfax University, Sfax 3029, Tunisia; ahmed_hakim2002@yahoo.fr; 9 Interdisciplinary Laboratory in Neurosciences, Physiology and Psychology: Physical Activity, Health and Learning (LINP2-2APS), UFR STAPS, UPL, Paris Nanterre University, 92000 Nanterre, France; tarak.driss@parisnanterre.fr

**Keywords:** redox status, physical exercise, untrained healthy young adults, physiological responses, anaerobic, antioxidant

## Abstract

Currently, it is well accepted that physical exercise-induced oxidative stress may damage biological structures and impair cellular functions. However, it is still unclear which type of exercise results in the greatest oxidative stress responses among a healthy untrained population. The aim of the present study was to compare the acute oxidative stress response (i.e., 0 to 20 min) following different types of exercise (anaerobic, aerobic, and combined). Ten healthy, untrained males (19.5 ± 1.7 years) performed three randomized exercise bouts: anaerobic (30 s Wingate test), aerobic (30 min at 60% maximal aerobic power (MAP)) or combined (anaerobic and aerobic). Venous blood samples were collected before, as well as at 0 (P0), 5 (P5), 10 (P10), and 20 (P20) min after each session. Rates of malondialdehyde (MDA) and antioxidant activities (i.e., glutathione peroxidase (GPX), superoxide dismutase (SOD), glutathione reductase (GR), α-tocopherol, and total antioxidant status (TAS)) were assessed. Independent of exercise type, plasma MDA, GPX, SOD, and GR contents increased above baseline, whereas plasma α-tocopherol decreased under baseline after the test sessions (*p* < 0.05). Aerobic and anaerobic exercises generated faster responses (at P0) when compared to the combined exercise (P5 to P10) for the majority of the tested parameters. Plasma TAS content only increased following the aerobic exercise at P10 (*p* = 0.03). Five to twenty-minutes post exercise, the highest MDA response was registered in the aerobic condition, and the highest GPX and SOD responses were recorded in the anaerobic (at P5) and aerobic (at P20) conditions (*p* < 0.05). In conclusion, aerobic, anaerobic, or combined exercises have the potential to acutely increase oxidative stress and antioxidant activities, but with different responses magnitude. These findings confirm that oxidative stress response seems to be dependent on the intensity and the duration of the physical exercise and may help in understanding how varying exercise bouts influence the degree of oxidative stress among healthy untrained young adults.

## 1. Introduction

Oxidative stress is characterized by the imbalance between pro-oxidant and antioxidant status, with the former outweighing the latter [1]. This imbalance can lead to physiopathological effects by increasing cells and cellular components’ (i.e., membranes, lipids, proteins, deoxyribonucleic acid (DNA), and lipoproteins, among the others) vulnerability to reactive oxygen species (ROS) attacks [1,2]. If not strictly controlled and counteracted, oxidative stress can cause acute pathologies (i.e., trauma and stroke) and be responsible for the insurgence of several chronic and degenerative diseases [3]. In order to properly protect the cells against the harmful effects of free radicals, the human body is able to mount a cascade of defense mechanisms, including preventative, repairing, and scavenging ones, as well as through the amelioration of antioxidant activities [4,5].

It is well accepted that physical exercise is an activity that increases ROS production via increased phospholipase A2 (PLA2), nicotinamide adenine dinucleotide phosphate (NADPH) oxidase, and xanthine oxidase (XO) activities, which, when combined, lead to oxidative stress [5,6,7,8]. During strength-based exercises [5,6,9], short-term maximal sprints [10,11], and exercise performed near the anaerobic threshold [12], the level of lipid peroxidation significantly increases immediately and up to 48 h following the effort. The mitochondrial electron transport chain complex, the phenomenon of ischemia-reperfusion injury, and local inflammation have all been identified as major sources of free radical production and induced oxidative stress during exercise [13]. These acute changes in oxidative stress-related biomarkers following exercise are also accompanied by an increase in antioxidant responses. Indeed, immediate increases in the content of uric acid (UA), catalase (CAT), and glutathione peroxidase (GPX) are registered following intensive strength, sprint, and Wingate efforts [14,15], with a return to baseline occurring from 10 min [16,17] to 4–8 h [5,6].

The aforementioned studies clearly describe intensive physical exercise as a situation resulting in oxidative stress, characterized by acute and delayed redox imbalance (i.e., between pro-oxidants and antioxidants). However, it is still unclear which type (aerobic, anaerobic, or combined anaerobic plus aerobic) of exercise results in the greatest oxidative stress responses [18,19]. For instance, limited investigations have explored the effects of exercises’ type on redox balance [20,21,22,23,24,25]. These studies have been limited only on aerobic- and anaerobic-based exercises, with no firm conclusion. Indeed, Bloomer et al. [20,21,22] and Parker et al. [23,24] suggested aerobic exercise induces a greater increase in pro-oxidant status when compared to anaerobic exercise, and Inal et al. [25] and Marzatico et al. [26] demonstrated the activities of enzymatic antioxidant defense increase similarly following aerobic and anaerobic exercises, while Parker et al. [23,24] showed that increasing exercise intensity resulted in greater endogenous antioxidant defenses.

Discrepancies between findings may be attributable to participant training level. In this sense, it is well described that longitudinal steady state exercise [25,26], as well as an effective resistance training program [1,5,6,7,8,27,28], could prevent or suppress increases in malondialdehyde (MDA) levels after physical efforts and reinforce the body’s defense against other oxidative attacks via activation of the redox sensitive transcription factors that finely regulate gene and protein expression within skeletal muscle and increased production of endogenous antioxidants (GPX, CAT, superoxide dismutase (SOD), and glutathione (GSH)) [19].

To the authors’ knowledge, there are only one study focused on the effect of three types of exercise (aerobic-based exercise, anaerobic based exercise, and combined exercise) on oxidative stress response [29]. This recent study was conducted by our research team and reported that redox-related biomarkers exhibited divergent response dynamics at 20 min following exercises with aerobic-based exercise generates greater MDA response, while anaerobic-based exercise generates lower antioxidant responses (e.g., GPX, SOD, glutathione reductase (GR), and total antioxidant status (TAS)) compared to the two other type of exercises.

However, given that this study investigated only well-trained athletes and given that training level was shown to be a disruptive factor in previous studies, it was suggested that the practical application of these preliminary findings is limited to a trained population, and more research is needed to corroborate it in a non-athletic population. Therefore, the aim of the present study was to compare levels of lipid peroxidation and antioxidant biomarkers immediately and up to 20 min following anaerobic, aerobic, or combined (anaerobic and aerobic) exercise performed by healthy untrained young adult males. Resolving which exercise type elicits the greatest oxidative stress response in healthy untrained subjects is important as this will help with understanding of redox homeostasis and preventing the harmful effect of exercise-induced oxidative stress among this population.

## 2. Materials and Methods 

### 2.1. Participants Selection: Inclusion and Exclusion Criteria

Ten healthy untrained males (19.5 ± 1.7 years, 71.8 ± 2.1 kg, 1.76 ± 0.17 m (mean ± SD)) volunteered to participate in this study. The participants were recruited on the basis that they had not participated in any type of regular physical training for at least one year before the experiment, as measured using the “International Physical Activity Questionnaire” and that they were not suffering from any kind of acute or chronic diseases and any kind of injury within three months of the start of the experiment.

To avoid any possible bias related to nutrition-derived assets of antioxidants (e.g., different nutrients’ protection levels), participants were instructed to avoid the consumption of any medications (e.g., antioxidant or anti-inflammatory drugs) or dietary supplements (e.g., creatine, foods rich in antioxidants or polyphenols, such as blueberries, coffee, green tea, grapes, cherries, curcuma, red wine, and dark chocolate) during the experimental period and for at least 8 weeks before the commencement of the study.

### 2.2. Ethical Clearance

Participants were informed of all procedures, potential risks, and benefits associated with the study and they provided written informed consent to take part in the experiment. The study was conducted according to the declaration of Helsinki, and the protocol was fully approved (identification code: 8/16) by the university institutional review board before the commencement of the assessment.

### 2.3. Experimental Design

One week before the start of the experimental period, VO_2 peak_ and maximal aerobic power (MAP) output was determined for each participant from an incremental laboratory cycling test [29,30]. After a 10 min warm-up at 100 W, the test began at an initial power output of 200 W. Subsequently, power output was increased by 30 W every 4 min until respiratory exchange ratio (RER) ⩾ 1. Thereafter, power output was increased by 10 W/min until exhaustion. During the test, VO_2_ was measured breath by breath using an indirect calorimetry system (Quark PFT, Cosmed, Rome, Italy) [29,30]. MAP was calculated using the equation proposed by Kuipers et al. [31]. The VO_2 peak_ was determined from the mean VO_2_ over the last 30 s of the test [29].

As part of a repeated-measures, cross-over experimental design, participants performed three randomized test sessions, with a recovery period of 72 h in between. To avoid any chronobiological effects [5,7,32,33,34], all test sessions were performed at the same time of day (around 08.00 hours). 

The test sessions consisted of either anaerobic-based (i.e., 30 s standard Wingate test), aerobic-based (i.e., 30 min low-intensity pedaling exercise), or combined (aerobic and anaerobic) exercise. Upon arrival for their first test session, each participant’s body mass (Tanita, Tokyo, Japan) and height were recorded. Before completing the experimental testing sessions, a standardized 5 min cycling warm-up was completed at 75 W. The anaerobic-based protocol comprised a single standard 30 s Wingate test on an electronically-braked cycle ergometer (Excalibur Sport, Lode B.V, Medical Technology, Groningen, Netherlands) connected to a computer with diagnostic software (Ergocard^®^, Medisoft, Dinant, Belgium). Following the warm-up, participants were instructed to pedal as fast as possible during a 6 s acceleration phase to attain peak cadence [29]. Immediately following the acceleration phase, the load was electronically applied to the flywheel and subjects pedaled “all-out’’ for the entirety of 30 s [29]. The aerobic-based protocol consisted of pedaling on the same cycle ergometer at an intensity equal to 60% of MAP output for a duration of 30 min at a cadence of 60 rpm [29]. The combined protocol involved the completion of the anaerobic-based protocol followed by the aerobic-based protocol with 3 min passive recovery between these protocols. Before and after (i.e., at 0 min (P0), 5 min (P5), 10 min (P10), and 20 min (P20)) each training session, blood samples were collected from a forearm vein (dominant arm) through an intravenous cannula.

### 2.4. Dietary Records

To assess the adequacy and consistency of nutrient intake, a daily dietary record was completed over seven days. All participants received detailed verbal and written instructions on the process of recording their diet. Participants were asked to continue with their usual dietary habits during the period of dietary recording and to be as accurate as possible in recording the amounts and types of food and fluid consumed. A list of common household measures (e.g., tablespoons, cups) and specific information about the quantity in each measurement (grams, etc.) was given to each participant. The individual’s diet was evaluated using the Bilnu 4 software (SCDA Nutrisoft, Cerelles, France), and the food composition tables published by the Tunisian National Institute of Statistics in 1978. Estimated nutrient intakes were compared to reference dietary intakes for physically active people, and the daily nutriment data showed that total calorie, macronutrient, and micronutrient intakes were within the reference dietary intakes for healthy Tunisian adults, with no significant differences between the three test sessions (e.g., test session 1: 2875 ± 365 kcal/day; test session 2: 2798 ± 402 kcal/day; 2906 ± 438 kcal/day).

### 2.5. Blood Analysis

To eliminate inter-assay variance, all samples were analyzed in the same assay run. All assays were performed in duplicate in the same laboratory, with simultaneous use of commercially assay kits from Randox (Randox Laboratories Limited, 55 Diamond Road, Crumlin, County Antrim, BT29 4QY, United Kingdom). SOD, GPX, glutathione reductase (GR), and total antioxidant status (TAS) were measured using standard colorimetric assays (Randox Laboratories Limited, 55 Diamond Road, Crumlin, County Antrim, BT29 4QY, United Kingdom) as described by El Abed et al. [16,29]. Intra- and inter-assay coefficient of variation for the SOD were 0.8% and 0.9%; intra- and inter-assay coefficient of variation for the GPX were 0.9% and 1.0%; intra- and inter-assay coefficient of variation for the GR were 0.7% and 0.8%; and intra- and inter-assay coefficient of variation for the TAS were 0.6% and 0.7%.

α-tocopherol was extracted with hexane from human plasma and then measured via high performance liquid chromatography (HPLC). For specimen preparation, 100 µLof internal standard was mixed with100 µL of plasma in a 1.5 mL Eppendorf tube and vortexed for 5 s. Subsequently, 200 µL of ethanol was added and vortexed for 30 s, followed by 500 µL of Hexane and a further 1 min vortex. The mixture was centrifuged at 4000 rpm and 4 °C for 8 min with 450 µL of the supernatant removed and evaporated to dryness under a stream of nitrogen at room temperature.

Solids were extracted via the addition of 250 µL of methanol, followed by a 30 s vortex and the same centrifugation protocol described above, before being analyzed using the HPLC method described by Siluk et al. [35]. Intra- and inter-assay coefficient of variation for the a-tocopherol were 1.1 and 1.2%

MDA was assayed as a marker of lipid peroxidation using a colorimetric reaction, which uses 1-methyl-2-phenylindole as chromogen. 

Condensation of one molecule of MDA with 2 molecules of 1-methyl-2-phenylindole (MPI) under acidic conditions results in the formation of a chromophore with an absorbance maximum at 586 nm. A 7.6 mM solution of MPI was prepared immediately prior to use in 33% methanol in acetonitrile. A 650 μL aliquot of MPI was placed in each test tube, to which was added a solution of 200 μL of plasma. 

The tubes were well mixed, and 150 μL of 10 M HCl was added. After mixing once more, the tubes were sealed, and incubated for 60 min at 45 °C. After incubation, the tubes were chilled in an ice bath and spun at 10,000× *g* for 5 min, in order to fully remove debris. The absorbance at 586 nm was measured and subtracted from the blank value, obtained by replacing plasma with water. A calibration graph was prepared using 4 μmol/L, 8 μmol/L, 16 μmol/L, and 20 μmol/L of 1,1,3,3-tetramethoxypropane in 20 mM Tris-HCl, buffer, pH 7.4. Intra- and inter-assay coefficient of variation for the MDA were 1.6 and 1.7%.

### 2.6. Statistical Analysis

All statistical analyses were performed using the commercial statistical software STATISTICA (StatSoft, Paris, France, version 10.0). Normality of the data distribution was confirmed using the Shapiro-Wilks-W-test. Values were computed and reported as mean ± SEM (standard error of the mean).

The data obtained for all antioxidants and oxidative stress markers were analyzed using a two-way ANOVA (3 levels (exercise type (anaerobic, aerobic, and combined—aerobic and anaerobic)) × 5 levels (samples-time (before, P0, P5, P10, and P20))) with repeated measure. Fisher’s least significant difference (LSD) *post-hoc* tests were conducted when a statistically significant main effect was found. Effect sizes were calculated as partial eta-squared (*η_p_^2^*) to assess the potential practical significance of the findings. 

For all analyses, statistical significance was set at *p* < 0.05.

## 3. Results

**Figure 1:**
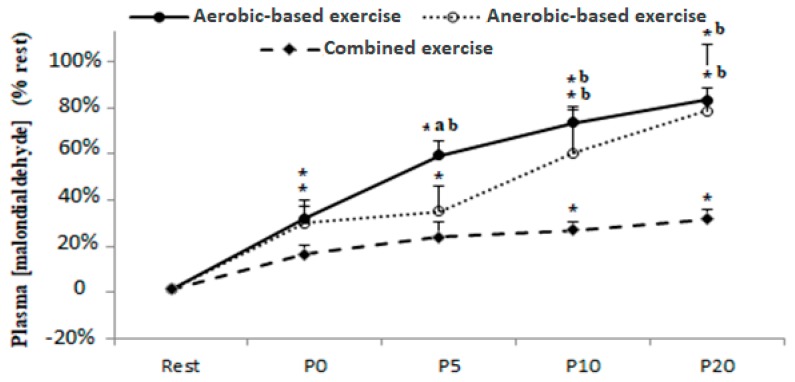
Plasma malondialdehyde concentration before (Rest), immediately after (P0), and 5 (P5), 10 (P10), and 20 (P20) min after aerobic, anaerobic, and combined (anaerobic and aerobic) exercise. Data are expressed as the % change from pre-exercise resting concentrations. *, **: significant difference when compared to pre-test values at the level of *p* < 0.05 and *p* < 0.01 espectively; a: significant difference when compared to the anaerobic exercise; b: significant difference when compared to the combined (anaerobic and aerobic) exercise.

The levels of lipid peroxidation at pre-test and at P0, P5, P10, and P20 following aerobic, anaerobic, and combined (anaerobic and aerobic) exercise are presented in Figure 1.

Statistical analysis showed statistically significant main effects of samples-time and exercise-type with *F*(4,36) = 24.84, *p* = 0,000, *η_p_^2^* = 0.28, and *F*(2,18) = 4.62, *p* = 0.03, *η_p_^2^* = 0.32, respectively.

Plasma MDA increased immediately (P0) after the aerobic and anaerobic test sessions when compared to the resting baseline with *p* = 0.02. However, MDA content did not increase above the resting baseline in the combined (anaerobic and aerobic) condition until 10 min post exercise (*p* = 0.04).

Moreover, MDA was higher at P5 post aerobic exercise when compared to the anaerobic (*p* = 0.05) and combined (anaerobic and aerobic) exercise (*p* = 0.005) and higher at P10 and P20 following the same exercise when compared to the combined (anaerobic and aerobic) condition with *p* = 0.000.

**Figure 2:**
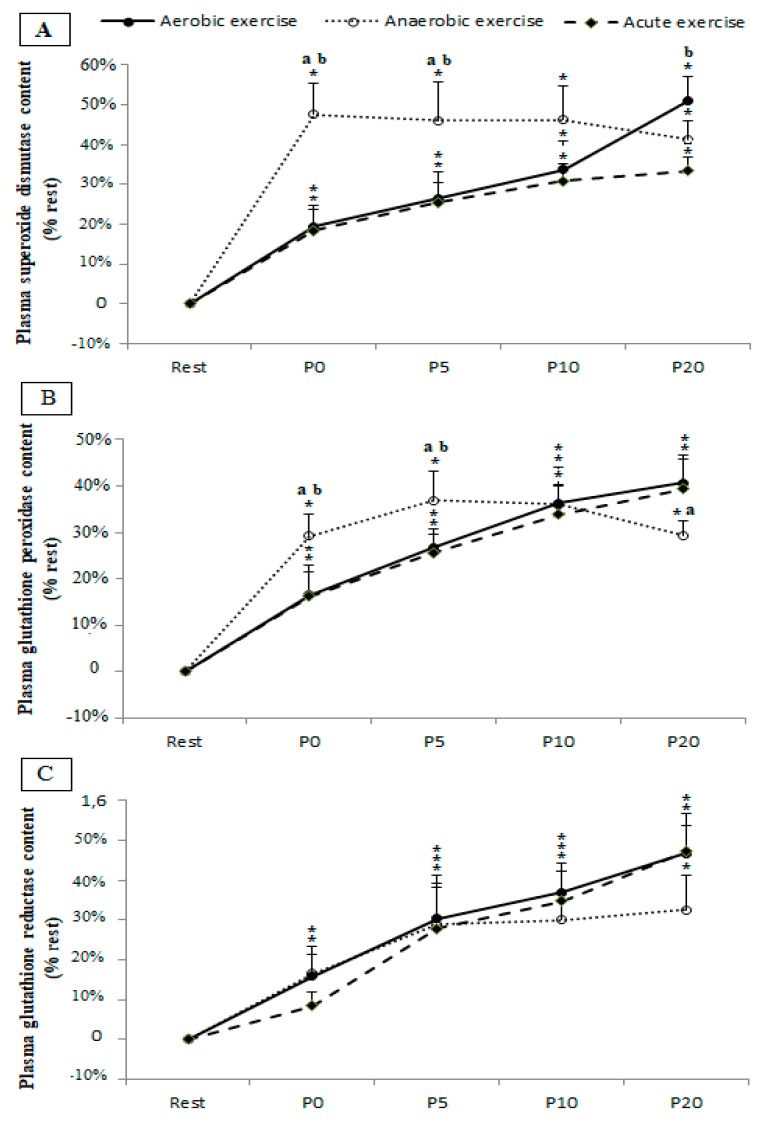
Superoxide dismutase (panel **A**), plasma glutathione peroxidase (panel **B**), and glutathione reductase (panel **C**) content before (Rest), immediately after (P0), and 5 (P5), 10 (P10), and 20 (P20) min after aerobic, anaerobic, and combined (anaerobic and aerobic) exercise. Data are expressed as the % change from pre-exercise resting concentrations. *, **, ***: significant difference when compared to pre-test values at the level of *p* < 0.05, *p* < 0.01 and *p* < 0.001 respectively; a: significant difference when compared to the aerobic exercise; b: significant difference compared to the combined (anaerobic and aerobic) exercise.

Figure 2 shows the enzymatic antioxidant responses following the different exercises types. A significant main effect of samples-time was registered for SOD (*F*(4,36) = 19.38, *p* = 0.000, *η_p_^2^* = 0.68), GPX (*F*(4,36) = 25.40, *p* = 0.000, *η_p_^2^* = 0.74), and GR (*F*(4,36) = 16.04, *p* = 0.000, *η_p_^2^* = 0.64).

Regardless of exercise type, the plasma content of SOD and GPX increased immediately (P0) post-exercise (*p* = 0.02, *p* = 0.000 and *p* = 0.03 for SOD and *p* = 0.004, *p* = 0.000, and *p* = 0.004 for GPX at P0 post aerobic, anaerobic, and combined (anaerobic and aerobic) exercise, respectively).

However, an immediate (P0) increase of GR content was only registered following the aerobic (*p* = 0.048) and anaerobic exercise (*p* = 0.039), while this content was not increased above the resting baseline in the combined (anaerobic and aerobic) condition until 5 min post exercise (*p* = 0.000).

When compared to the aerobic and combined (anaerobic and aerobic) conditions, anaerobic exercise resulted in greater SOD and GPX levels at P0 (*p* = 0.000 for SOD and *p* = 0.02 for GPX) and P5 (*p* = 0.02 for SOD and *p* = 0.05 for GPX).

At P20 greater SOD levels were registered following the aerobic exercise when compared to the combined (anaerobic and aerobic) condition (*p* = 0.03), and greater GPX levels were registered following the same exercise when compared to the anaerobic condition (*p* = 0.04).

**Figure 3:**
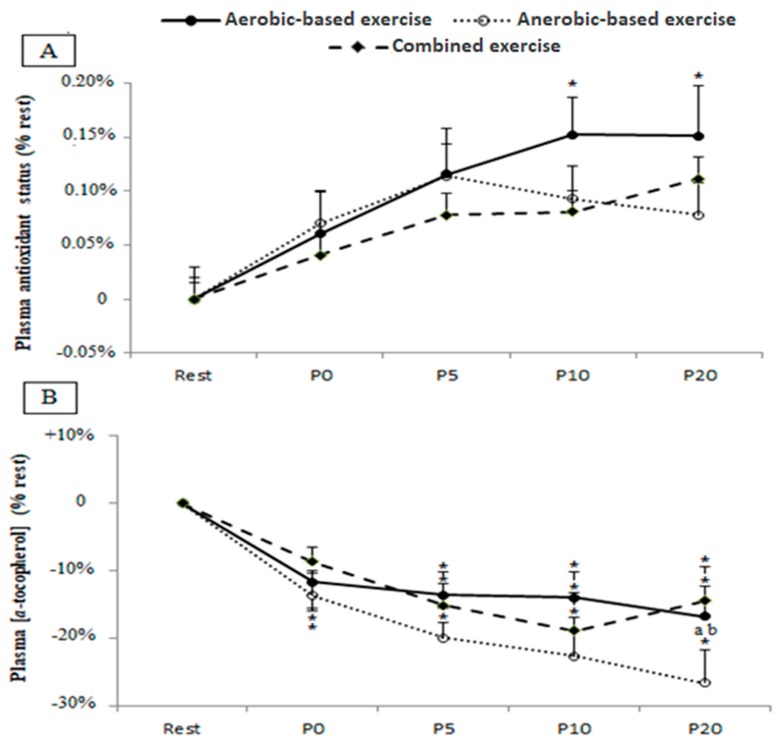
Plasma antioxidant status (panel **A**) and α-tocopherol concentration (panel **B**) before (Rest), immediately after (P0), and 5 (P5), 10 (P10), and 20 (P20) min after aerobic, anaerobic, and combined (anaerobic and aerobic) exercise. Data are expressed as the % change from pre-exercise resting concentrations. *: significant difference when compared to pre-test values; a: significant difference when compared to the aerobic exercise; b: significant difference when compared to the combined (anaerobic and aerobic) exercise.

Plasma TAS and α-tocopherol following the different exercise protocols are shown in Figure 3. There was a statistically significant main effect for samples-time for TAS (*F*(4,36) = 3.2, *p* = 0.025, *η_p_^2^* = 0.26), and α-tocopherol (*F*(4,36) = 15.15, *p* = 0.000, *η_p_^2^* = 0.63).

A significant increase of TAS values from pre- to post-exercise was only registered following the aerobic exercise at P10 post-exercise with *p* = 0.03.

However, plasma α-tocopherol decreased immediately (P0) after the aerobic (*p* = 0.02) and anaerobic (*p* = 0.005) test sessions when compared to the resting baseline, while this decrease was not registered in the combined (anaerobic and aerobic) condition until 5 min post-exercise (*p* = 0.002).

When compared to the aerobic and combined (anaerobic and aerobic) exercise, lower content of α-tocopherol was registered in the anaerobic condition at P20 with *p* = 0.04 and *p* = 0.01, respectively.

## 4. Discussion

The aim of the present study was to compare the acute oxidative stress response (0 to 20 min) following anaerobic, aerobic, or combined (anaerobic and aerobic) exercise among healthy untrained young adult males. Immediately following aerobic and anaerobic exercise, an increased level of plasma MDA, SOD, GPX, and GR and a decreased level of plasma α-tocopherol were registered, while immediately following the combined (anaerobic and aerobic) exercise, only an increased level of GPX and SOD was registered. Plasma TAS increased following only the aerobic exercise at P10. Most importantly, original findings of the current study showed the greatest MDA response starting at 5 min and continuing on following the aerobic exercise and greatest SOD and GPX responses at P0 and P5 post anaerobic based-exercise and at P20 post aerobic exercise.

These findings offer insight into exercise-type-specific oxidative stress development in healthy untrained young adult populations. The effect of exercise-type on the oxidative stress response is still under debate. Indeed, following aerobic exercise some authors have found a significant increase in oxidative stress response [10,29,30], while other studies have reported no changes in MDA levels from pre- to post-exercise [36,37,38]. Similarly, different findings were shown during combined (anaerobic and aerobic) [10,29,39,40] or anaerobic [10] exercise, with either a significant MDA increase pre-post exercise [10,29,39,40] or no significant effect of the physical exercise [41].

Discrepancy between results could be attributed to methodological issues, such as the diversity of assays used to evaluate lipid peroxidation products or/and to the training level of participants, with well-trained subjects having more reinforced defense against oxidative attacks [42]. For future investigations, investigating healthy untrained populations and analyzing the same oxidative stress parameters within the same test conditions can help resolve the contrasting findings from previous investigations.

The present results confirm the fact that physical exercise (i.e., aerobic, anaerobic or combined—anaerobic and aerobic) is a condition that results into oxidative stress [10,29,36,39,40] and specifically suggest that aerobic and anaerobic exercises induce higher oxidative stress when compared to combined (anaerobic and aerobic) exercise with greatest responses following the aerobic exercise at P5 post-exercise. These findings are consistent with previous findings in trained subjects showing that aerobic exercise induces a greater increase in pro-oxidants than anaerobic exercise, suggesting oxidative stress responses are dependent on exercise mode (i.e., intensity and duration) [29]. Exercise-induced oxidative stress has been attributed to ROS generated through enzymatic and non-enzymatic processes. Enzymatic sources include NADPH oxidases located on the cell membrane of polymorphonuclear cells, macrophages and endothelial cells [43,44], and cytochrome P450-dependent oxygenases [45], while the non-enzymatic source is related to the production of superoxide anion (O_2_^−^). The latter occurs when a single electron is directly transferred to oxygen by reduced coenzymes or prosthetic groups or by xenobiotics previously reduced by certain enzymes [46].

In the present study, the greatest oxidative stress response following the aerobic exercise at 5 min post-exercise could be explained by the higher activity of the mitochondrion (due to the higher oxygen consumption) during such exercise compared to anaerobic or combined exercise. Besides producing necessary adenosine triphosphate (ATP) during and following aerobic exercise, mitochondria also appear to be the main intracellular source of pro-oxidants. In fact, the mitochondrial electron transport chain contains several redox centers that may leak electrons to oxygen and reduce it to O_2_^−^ that is involved in the propagation of oxidative chain reactions, which is a precursor of other ROS [46].

Concerning the antioxidant defense, it was previously suggested that, in response to an increased production of free radicals, increased concentrations of antioxidant enzymes may occur to counteract the radical production and minimize oxidative damage. The present findings confirm this suggestion showing significant increases pre-post exercise for the enzymatic defense (i.e., GPX, SOD, and GR), regardless of the type of exercise. Additionally, these findings are in line with previous studies reporting increased enzymatic antioxidant activities immediately following (1) anaerobic exercise, such as strength eccentric exercise [47] and 100 m swim [25], (2) aerobic exercise, such as low intensity running [27,28] or swimming [25] activities, and combined (anaerobic and aerobic) exercise, such as intermittent (6 × 150 m) sprints [26]. Authors of these studies also attributed increases in these enzymatic antioxidant levels to the increase of the oxygen consumption, acidosis, catecholamines, and xanthine oxidase activity. Most importantly, present findings suggest the majority of changes in plasma contents of antioxidant parameters appeared immediately (P0) following aerobic and anaerobic exercise and at 5 min following the combined (anaerobic and aerobic) exercise.

These results suggest that the response of antioxidant-mechanism may take place 0 to 5 min following the physical effort, albeit this is dependent on exercise type. This duration has also been dependent on the time-kinetics of free radical production [27,28] and proportional to the intensity of exercise [48]. The present observations confirm these suggestions and showed similar magnitude of lipid peroxidation and antioxidant responses to physical exercise with faster change in aerobic and anaerobic conditions (at P0) when compared to combined (anaerobic and aerobic) one (P5 to P10).

A previous study in athletic populations showed levels of enzymatic antioxidants were returned to baseline values between 10- and 20 min post-exercise [16]. In contrast, using untrained populations, the present study showed none of the tested parameters returned to baseline level at 20 min post-exercise. The low training status of the involved subjects may explain these results. Indeed, being well trained could induce an activation of the redox sensitive transcription factors (e.g., nuclear factor-kappa B (NFkB)) that improve the production of endogenous antioxidants, accelerating their protective effect against ROS production [49] in response to physical exercise. Concerning the TAS and the non-enzymatic antioxidant responses, the present results showed a significant increase of plasma TAS content following only the aerobic exercise (P10) and a significant decrease in α-tocopherol levels following the three types of exercises with faster change in aerobic and anaerobic conditions.

These results are in line with previous studies reporting decreased α-tocopherol levels following anaerobic [14] and combined [16] exercises and increased TAS levels following aerobic [50] and combined (anaerobic and aerobic) [16] exercises. The increased level of TAS could be attributed to increased levels of uric acid [50].

### Limitations and Future Prospects

Given that TAS essentially measures the effectiveness of water-soluble antioxidants, such as uric acid, albumin, thiols, or vitamin C [51], including a measure of uric acid in future investigations, is needed to well understand the kinetic-response of TAS.

Similarly, the measurement of concentrations of other plasma nonenzymatic antioxidants (vitamin C, vitamin A, GSH) could complement the measure of a-tocopherols and may provide further information concerning the effect of exercise mode on the antioxidant status.

Finally, the present study was designed as a pilot study with small number of participants, which makes such a study highly vulnerable to biases introduced by individual lifestyle and history. Therefore, future investigations involving larger sample sizes and females are warranted.

## 5. Conclusions

The results of this study demonstrate, in a small sample of healthy untrained young adults, anaerobic, aerobic, or combined (anaerobic and aerobic) exercise can alter antioxidant status as a response to the increased lipid peroxidation with (1) faster responses occurring during the aerobic and anaerobic conditions (at P0), (2) greater level of MDA generated following 5 min of the aerobic exercise, and (3) greater levels of SOD and GPX generated during the anaerobic (at P0 and P5) and aerobic conditions (P20).

These observations support, in the present investigated untrained population, the development of exercise-induced oxidative stress but, most importantly, showed the magnitude and dynamics of oxidative stress appearance/development to be dependent on the intensity and duration of exercise.

These pilot findings suggest insight into how different types of exercise influence the degree of oxidative stress responses among healthy untrained young adult population. However, a larger study taking into consideration individual lifestyle variables is needed to corroborate these findings.
